# Influence of Infill Density on Bacterial Colonization and Mechanical Performance of 3D-Printed PETG Scaffolds

**DOI:** 10.3390/polym18182299

**Published:** 2026-09-20

**Authors:** Raghad A. Alabdli, Rewaa S. Jalal, Abdulrahman A. Alqarni, Ahmad A. Basalah, Laila A. Damiati

**Affiliations:** 1 Department of Biological Sciences, Collage of Science, University of Jeddah, Jeddah 21589, Saudi Arabia; 2 Mechanical Engineering Department, College of Engineering, King Khalid University, Abha 61421, Saudi Arabia; 3 Mechanical Engineering Department, Collage of Engineering and Architecture, Umm Al-Qura University, Makkah 21955, Saudi Arabia

**Keywords:** PETG, 3D printing, infill density, tensile test, antibacterial application

## Abstract

Polyethylene terephthalate glycol (PETG) is a versatile thermoplastic widely used in food packaging and biomedical applications owing to its excellent mechanical properties, chemical resistance, biocompatibility, and ease of processing. Porosity is a critical design parameter that governs the biological and mechanical performance of 3D-printed PETG structures by influencing cell attachment, nutrient transport, mechanical integrity, and microbial interactions. In this study, PETG scaffolds were fabricated at five nominal infill densities (20%, 40%, 60%, 80%, and 100%) using fused deposition modelling (FDM) to investigate the influence of printing-defined architecture on mechanical performance and bacterial colonization. Tensile testing revealed that decreasing infill density resulted in a progressive reduction in ultimate tensile strength and Young’s modulus, demonstrating the trade-off between reduced material density and mechanical integrity. Bacterial interactions with the scaffolds were evaluated against *Escherichia coli*, *Pseudomonas aeruginosa*, *Staphylococcus aureus*, *Staphylococcus epidermidis*, and *Salmonella typhimurium* using optical density (OD) and ATP-based viability assays to quantify both planktonic and adherent bacterial populations. The results demonstrated that the influence of scaffold porosity was species-dependent. The results demonstrated species-dependent responses to scaffold architecture. Higher infill densities were generally associated with reduced attachment and metabolic activity for *P. aeruginosa*, *S. epidermidis*, and *S. typhimurium*, whereas *E. coli* exhibited relatively stable growth and metabolic activity across the tested infill conditions. *S. aureus* showed an intermediate response, with bacterial attachment and metabolic activity varying according to scaffold architecture. Overall, infill density exerted a greater influence on scaffold-associated bacteria than on planktonic populations, while simultaneously determining the mechanical performance of the printed structures. These findings demonstrate that controlling PETG infill architecture is important for balancing mechanical properties and bacterial colonization and provide design insights for future PETG-based biomedical and food-packaging applications.

## 1. Introduction

Polyethylene terephthalate glycol (PETG) is a glycol-modified copolyester that has attracted considerable interest owing to its excellent mechanical strength, high ductility, optical transparency, and resistance to heat and chemical degradation. Compared with conventional polyethylene terephthalate (PET), PETG exhibits improved processability due to its amorphous structure and higher glass transition temperature (approximately 85 °C), making it an attractive material for additive manufacturing applications. These characteristics have led to its widespread use in food packaging, where durability, transparency, and food-contact safety are essential, as well as in biomedical applications, including tissue engineering scaffolds, pharmaceutical drug delivery systems, and medical devices for dental, orthopaedic, vascular, neurological, surgical, and ophthalmic applications [1,2,3]. Among the available additive manufacturing technologies, fused filament fabrication (FFF), also referred to as fused deposition modelling (FDM), is one of the most widely adopted techniques for processing PETG because of its simplicity, cost-effectiveness, and ability to fabricate complex three-dimensional structures with minimal material waste. Unlike conventional subtractive manufacturing, FDM builds components layer-by-layer, enabling precise control over scaffold geometry, pore size, and overall porosity while achieving high material utilization and producing near-net-shape structures [1]. These advantages have made FDM an attractive manufacturing technique for producing customized scaffolds for biomedical engineering and functional polymeric structures for industrial applications. The microarchitecture of tissue engineering scaffolds, particularly pore size, porosity, and pore interconnectivity, plays a crucial role in determining both their biological and mechanical performance [4,5,6]. Highly porous scaffolds facilitate cell infiltration, nutrient transport, vascularization, and tissue regeneration; however, increasing porosity often compromises mechanical strength [4,7,8]. This trade-off is particularly relevant for FDM-fabricated PETG structures because infill density directly determines the amount and distribution of material within the printed construct. Pervious work has similarly confirmed that infill density and internal architecture exert a strong influence on the tensile strength, stiffness, deformation behavior, and load-bearing capacity of FDM-printed PETG structures. Srinivasan et al. demonstrated that increasing PETG infill density from 20% to 100% produced a progressive increase in tensile strength, with the highest tensile strength reported for specimens printed at 100% infill density [9].

In addition to influencing mammalian cell behaviour, scaffold architecture also affects microbial adhesion and biofilm formation. Previous studies have demonstrated that pore geometry and surface topography significantly influence bacterial attachment by modifying the available surface area, nutrient diffusion, and bacteria–material interactions [4,5,6,7,8,9,10,11]. Cheng et al. showed that bacterial attachment can be highly sensitive to micro- and nanoscale topographical features because these features alter the effective bacteria–surface contact area and local physicochemical interactions. Likewise, subsequent reviews have highlighted that three-dimensional surface topography can either suppress or promote bacterial colonization depending on feature dimensions, spatial organization, and bacterial phenotype [12]. Surface patterning, roughness, and wettability have therefore increasingly been explored as non-chemical strategies for controlling bacterial adhesion and biofilm formation without necessarily incorporating conventional antimicrobial agents [13].

Furthermore, the layer-by-layer fabrication process inherent to FDM generates characteristic surface features that can alter bacterial colonization patterns on polymeric scaffolds. Consequently, optimizing scaffold architecture represents an important strategy for balancing biological performance, mechanical integrity, and resistance to bacterial contamination. Previous studies have demonstrated that variations in pore geometry and carrier architecture can alter microbial immobilization, biomass retention, nutrient accessibility, and biofilm development [14,15,16,17]. For instance, Widjaya et al. reported that modification of the pore geometry of additively manufactured PETG carriers altered biofilm attachment and microbial activity, supporting the concept that PETG architecture itself can regulate microorganism–material interactions. Furthermore, studies of 3D-printed PETG have shown that changes in infill density and internal geometry substantially modify structural and surface characteristics, which may consequently affect biological interactions [18].

Nevertheless, most previous studies on FDM-fabricated PETG have focused primarily on processing parameters, mechanical properties, surface roughness, biocompatibility, or individual biofilm-related applications. Comparatively little information is available on how systematically varied PETG infill architecture influences both planktonic and surface-associated bacterial populations across multiple clinically relevant bacterial species. Moreover, the relationship between architecture-dependent bacterial attachment and bacterial metabolic activity has rarely been examined together with the corresponding mechanical response of PETG structures. Addressing this gap is important because an architecture that improves biological transport or reduces bacterial attachment may simultaneously compromise mechanical integrity [19,20]. Therefore, the present study investigated FDM-fabricated PETG structures produced at five nominal infill densities (20%, 40%, 60%, 80%, and 100%) to determine how printing-defined architecture influences mechanical performance and bacterial interactions. Mechanical behavior was assessed by tensile testing, whereas bacterial growth, surface attachment, and metabolic activity were evaluated using optical-density and ATP-based assays against *Escherichia coli*, *Pseudomonas aeruginosa*, *Staphylococcus aureus*, *Staphylococcus epidermidis*, and *Salmonella typhimurium*. The novelty of this study lies in the combined evaluation of mechanical behavior, planktonic bacterial growth, surface-associated bacterial attachment, and metabolic activity across multiple bacterial species under systematically varied PETG infill conditions. These findings demonstrate that controlling PETG infill architecture is important for balancing mechanical properties and bacterial colonization and provide design insights for future PETG-based biomedical and food-packaging applications.

## 2. Materials and Methods

### 2.1. Surface Fabrication

Commercial unreinforced polyethylene terephthalate glycol (PETG) filament supplied by Push Plastic (Push Plastic, Springdale, AR, USA) was used in this study. The filament had a nominal diameter of 2.85 mm and a manufacturer-reported density of 1.27 g/cm^3^. According to the manufacturer, the filament was produced from virgin PETG resin. We printed the 3D models which are in a round disk shape of Ø 10.8 × 2.5 mm, and they were designed by Craftware Pro v1. 1. 4. 95 (Craftunique Kft., Budapest, Hungary). The models were sliced in Ultimaker Cura v4 and then exported in 2D layers to be G-coded for 3D printing purposes. An Ultimaker3 3D Printer (Utrecht, The Netherlands) was used for the 3D printing process.

**Figure 1:**
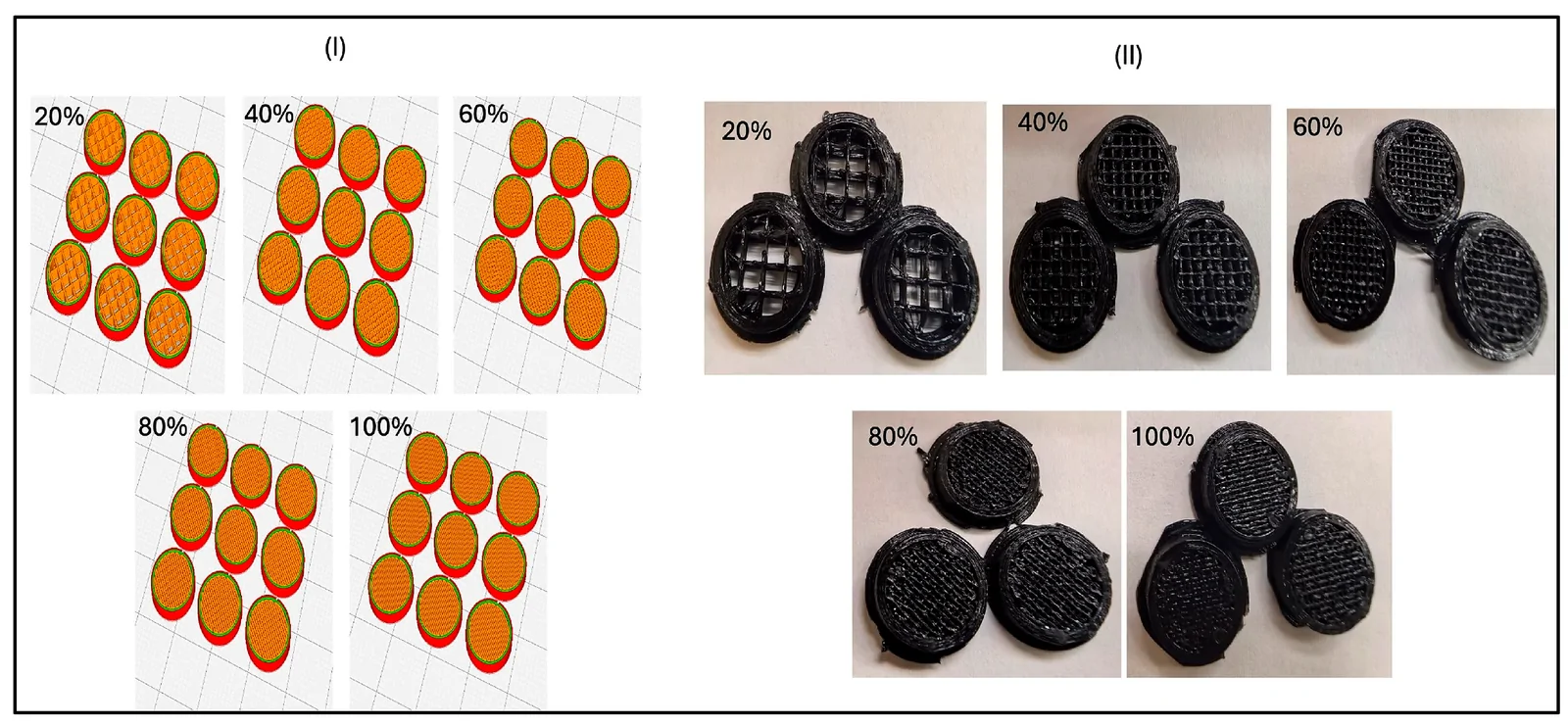
(**I**): CAD model of samples with different nominal infill densities. (**II**): Images of the actual scaffolds after printing (20%, 40%, 60%, 80%, 100%).

During printing, samples were fabricated at five nominal infill densities: 20%, 40%, 60%, 80%, and 100%. These levels were selected to provide a systematic range of printing-defined internal architectures, from structures with relatively high designed internal void space at 20% infill to fully filled structures at 100% infill. Infill density refers to the proportion of the internal volume occupied by the deposited PETG according to the slicer settings and should not be interpreted as experimentally measured scaffold porosity. Printing parameters were nozzle temperature of 240 °C, which falls within the processing range recommended by the filament manufacturer (235–250 °C). A 0.4 mm nozzle and a 0.2 mm layer height were selected to ensure consistent filament deposition and reproducible internal geometry; these settings are also in line with those used by the manufacturer for PETG characterization. The printing speed was set to 50 mm/s to maintain stable deposition while limiting dimensional variation and excessive fabrication time. The build-plate temperature was maintained at 70 °C to promote first-layer adhesion and reduce the risk of warping during printing (Figure 1).

### 2.2. Mechanical Test (Tensile Test)

**Figure 2:**
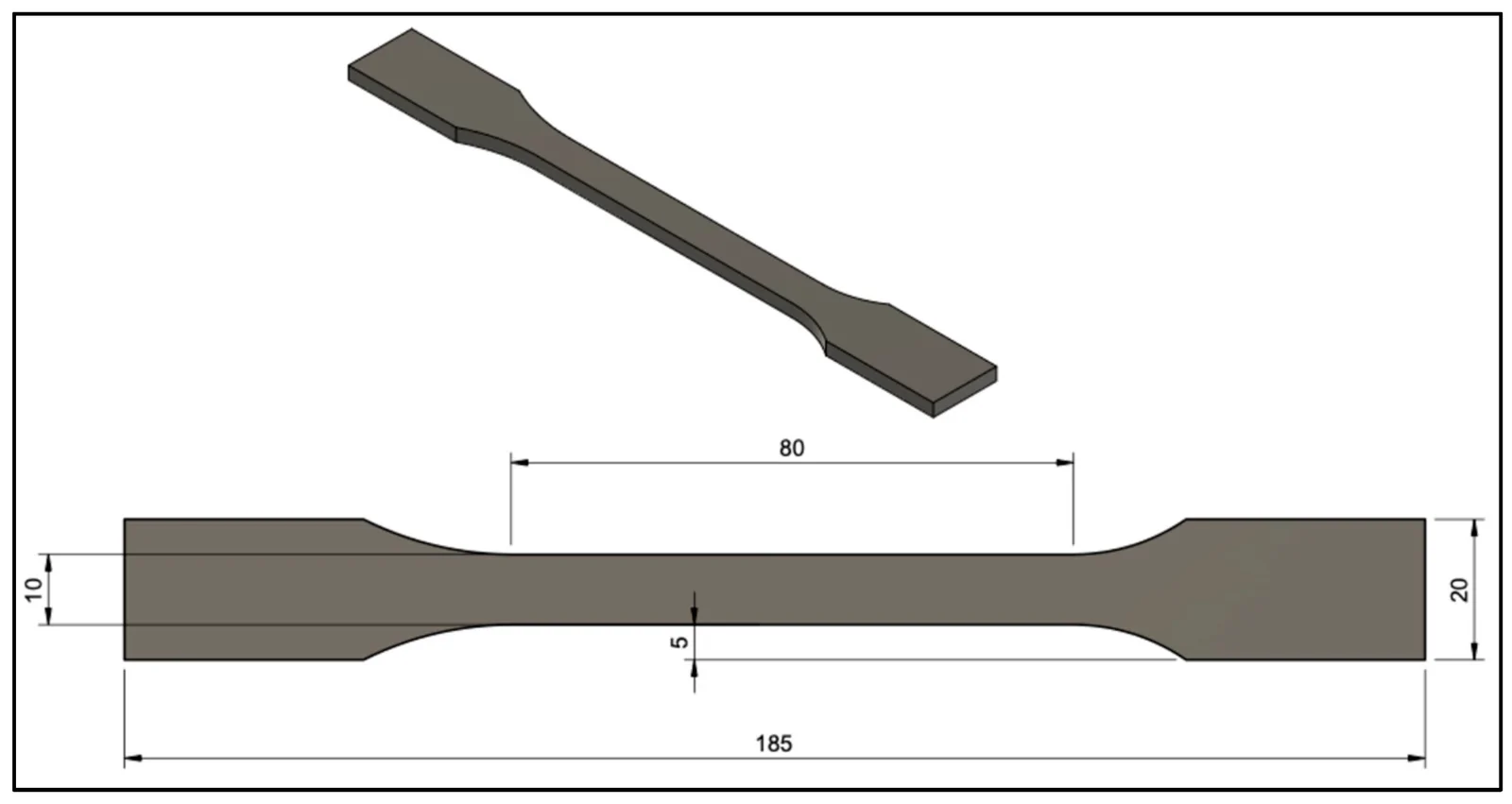
CAD model of the PETG tensile test specimen with key dimensions (in mm) following the ASTM D638 Type IV standard dog-bone geometry.

The tensile samples were designed according to the American Society for Testing and Materials D638 (ASTM) specifications for standard dog-bone coupons. As shown in (Figure 2), each specimen had an overall length of 185 mm with a gauge length of 80 mm. The narrow mid-section was ~10 mm in width and ~5 mm in thickness, while the end tabs broadened to 20 mm width to facilitate gripping.

Five nominal infill densities were investigated: 20%, 40%, 60%, 80%, and 100%. Infill density was controlled through the slicing software and represents the nominal proportion of the internal structure occupied by deposited PETG. Thus, 100% infill represents a fully filled structure with no intentionally designed internal voids, whereas progressively lower infill settings introduce increasing amounts of designed internal void space. The selected 20% increments provided a systematic range of internal architectures for evaluating the effects of infill density on mechanical and bacterial responses.

**Figure 3:**
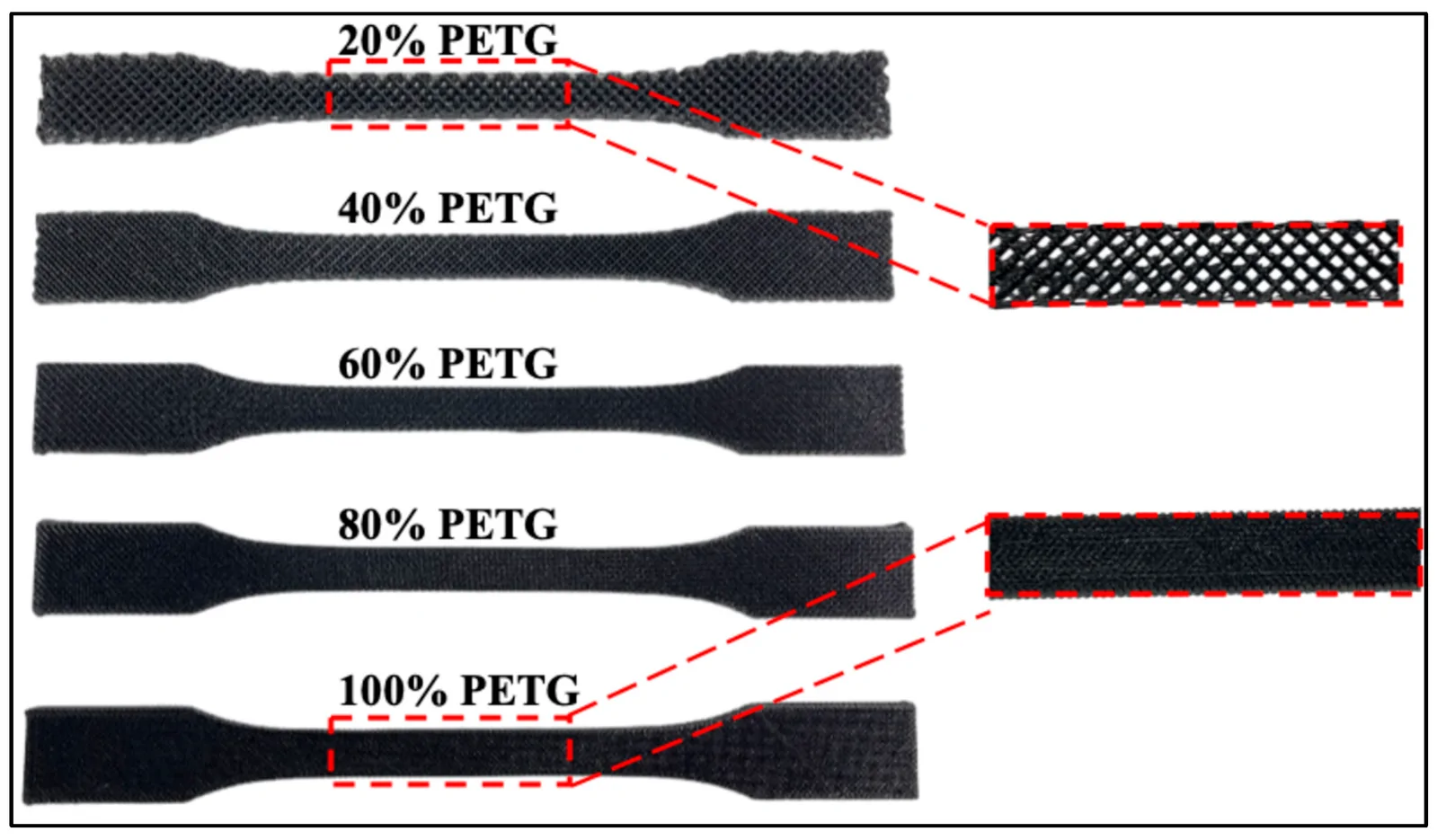
3D-printed PETG tensile specimens fabricated at different nominal infill densities (20%, 40%, 60%, 80%, and 100%), illustrating the progressive increase in the density of the internal printed structure with increasing infill percentage.

For each infill-density condition, at least five identical specimens were printed to provide replicates for testing. The as-printed specimens for each infill level are shown in (Figure 3), which illustrates the increasing density of the internal grid structure as the infill percentage rises from 20% to 100%.

Tensile tests were carried out in accordance with ASTM D638 to evaluate the mechanical properties of the PETG specimens. As previously mentioned, the printed dog-bone specimens (Figure 2) conformed to the standard dimensions so that they could be gripped and loaded uniformly. All tests were conducted on an Instron 3367 electromechanical universal testing machine (Instron^®^, Norwood, MA, USA) equipped with a 30 kN load cell. The test was performed ambient laboratory conditions (approximately 23 °C, 50% humidity). The specimen ends were gripped firmly, and axial strain was obtained from the crosshead displacement, corrected for machine compliance, as recorded by the testing software. Tensile loading was applied at a constant crosshead displacement rate of 1.0 mm/min, ensuring a quasi-static loading regime as prescribed by the standard. Ultimate tensile strength (UTS) for each specimen was calculated as the maximum load achieved before fracture divided by the original gross cross-sectional area of the gauge section, based on the external specimen dimensions. The cross-sectional area was not corrected for the internal void fraction associated with the different nominal infill densities. Young’s modulus was determined from the slope of the initial linear portion of the engineering stress–strain curve. All specimens were observed to fail within the gauge section, as intended; no slippage or grip failures occurred. The fracture surfaces and modes were noted for each sample to correlate with porosity. Overall, this testing procedure provided a consistent basis to compare the tensile behavior of PETG at different porosity levels under identical conditions.

### 2.3. Bacterial Culture

*Escherichia coli* (*E. coli*) (ATCC 25922), *Staphylococcus aureus* (*S. aureus*) (ATCC 12600), *Staphylococcus epidermidis* (*S. epidermidis*) (ATCC 29213), *Pseudomonas aeruginosa* (*P. aeruginosa*) (ATCC 1744), *Salmonella typhimurium* (*S. typhi*) (ATCC 13311) were cultured in a Muller-Hinton broth media and incubated overnight at 37 °C; for each design, scaffolds were placed in 24-well plates and seeded with 2 mL of bacterial suspension and incubated for 24 h at 37 °C. After 24 h, the scaffolds were transferred into 15 mL tube and then washed gently with phosphate-buffered saline (1× PBS), before being discarded. Afterwards, 1 mL of PBS was added to each tube containing the scaffolds and vortexed for 1 min. The optical density (OD) of adherent bacteria was measured at 600 nm using a UV–vis spectrophotometer (Thermo Scientific Genesys 10S UV–Vis, Waltham, MA, USA) while applying PBS as the blank. In addition, the OD of each scaffold’s supernatant was measured.

### 2.4. Bacterial Metabolic Activity Measurement (ATP Release)

Bacterial viability on the different PETG scaffolds was assessed using the BacTiter-Glo™ Microbial Cell Viability Assay (Promega, Southampton, UK). Porous PETG scaffolds were placed in 24-well plates and inoculated with a bacterial suspension at a concentration of 10^4^ CFU/mL. The plates were incubated under static conditions at 37 °C for 24 h. Following incubation, the supernatant containing planktonic (floating) bacteria was collected and transferred to 96-well plates for ATP quantification. The scaffolds were then gently washed with sterile 1× PBS to remove non-adherent bacteria. To recover adherent cells, the scaffolds were transferred to bijou tubes containing 1 mL of 1× PBS and sonicated for 10 min. The resulting bacterial suspensions were transferred to 96-well plates. An equal volume of BacTiter-Glo™ reagent was added to each sample, and luminescence was measured within 5 min using a SpectraMax^®^ i3 Multi-Mode Microplate Reader (Molecular Devices, San Jose, CA, USA). The luminescence signal, which is proportional to intracellular ATP levels, was used as an indicator of metabolically active bacterial biomass in the planktonic and scaffold-associated fractions.

### 2.5. Statistical Analysis

Experiments were performed as three independent replicates and the results were presented as means ± standard deviation (SDs). All statistical analyses were performed using GraphPad Prism V.11. Data were analyzed using Ordinary One-Way ANOVA, and *p*-values < 0.05 were considered significant.

### 2.6. Use of Artificial Intelligence-Assisted Language Editing

ChatGPT (OpenAI) was used during manuscript preparation solely to assist with English-language editing, including improvements in grammar, clarity, and readability. The tool was not used to generate experimental data, perform statistical analyses, interpret the results, or formulate scientific conclusions.

## 3. Results and Discussion

### 3.1. Effect of Infill Density on Tensile Behavior

**Figure 4:**
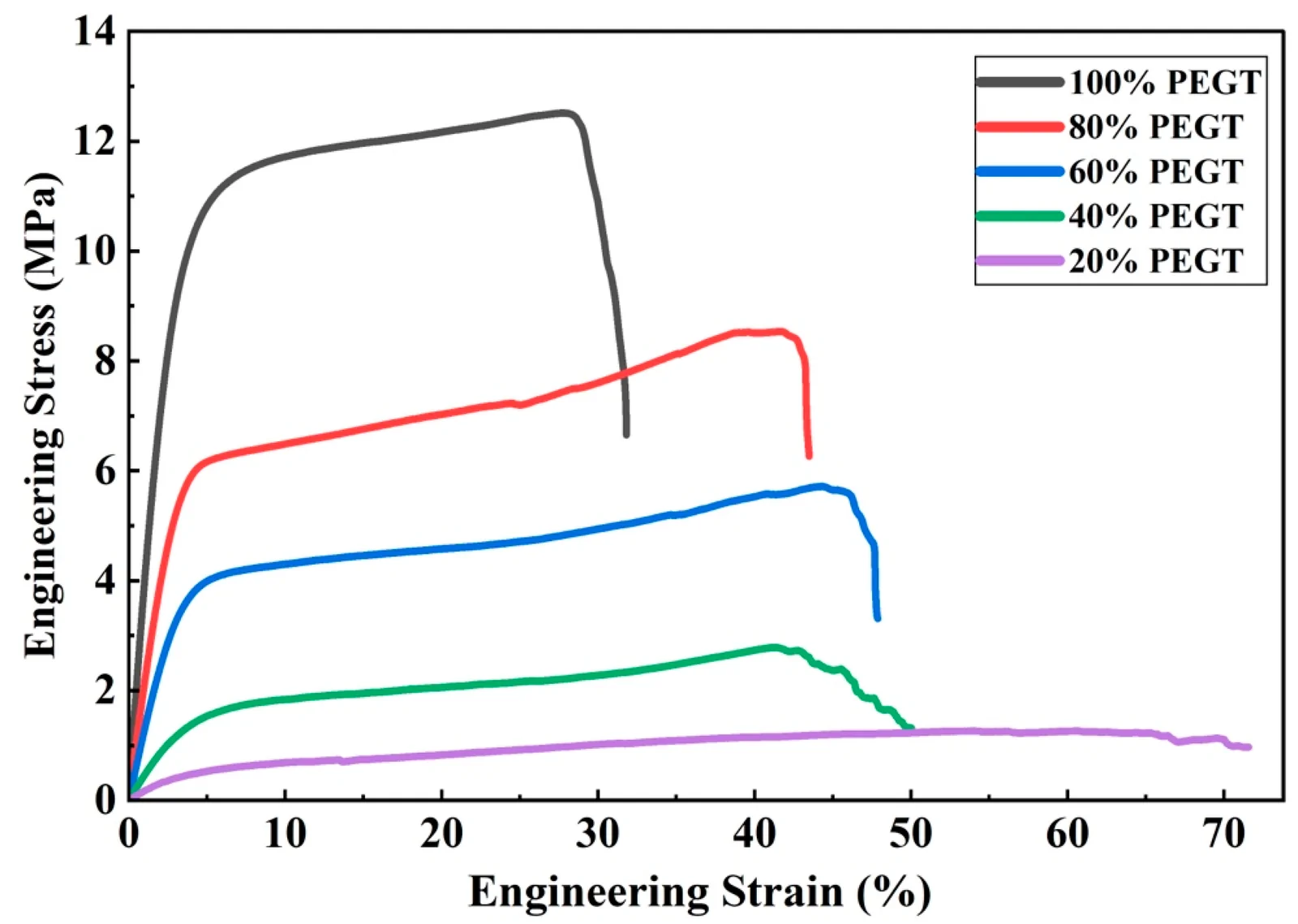
Engineering stress–strain curves for 3D-printed PETG tensile specimens at five infill/porosity levels (100%, 80%, 60%, 40%, 20%).

As shown in Figure 4, the tensile response of the PETG samples varies markedly with infill percentage. Tensile test was conducted in accordance with ASTM D638 (Type IV specimens). The engineering stress was calculated as

(1) $$\sigma =\frac{F}{A_{0}}$$

where F is the applied force and $A_{0}$ is the original cross-sectional area of the gauge section.

The initial tangent modulus ($E_{T}$) was determined from the slope of the stress–strain curve, where σ  represents engineering stress and ε  represents engineering strain:

(2) $$E_{T}={\frac{d\sigma }{d\varepsilon }|}_{\varepsilon \to 0}$$

The tensile response of the PETG specimens varied markedly with nominal infill density (Figure 4), with the corresponding mechanical properties summarized in Table 1. UTS increased progressively from 1.27 ± 0.15 MPa at 20% infill to 12.50 ± 0.75 MPa at 100% infill. A similar trend was observed for Young’s modulus, which increased from 18.5 ± 3.7 MPa at 20% infill to 418.0 ± 41.8 MPa at 100% infill, indicating a substantial increase in stiffness with increasing material content. In contrast, strain at fracture was generally greater at lower infill densities, reaching 74.0 ± 11.1% at 20% infill compared with 31.9 ± 3.2% at 100% infill. These findings demonstrate the architecture-dependent mechanical trade-off associated with infill density, whereby reducing the amount of load-bearing material decreases strength and stiffness while generally increasing deformation before fracture [9,10,11,12,13,14,15,16,17,18,19,20,21,22].

### 3.2. Effect of Infill Density on Bacterial Colonization and Metabolic Activity

**Figure 5:**
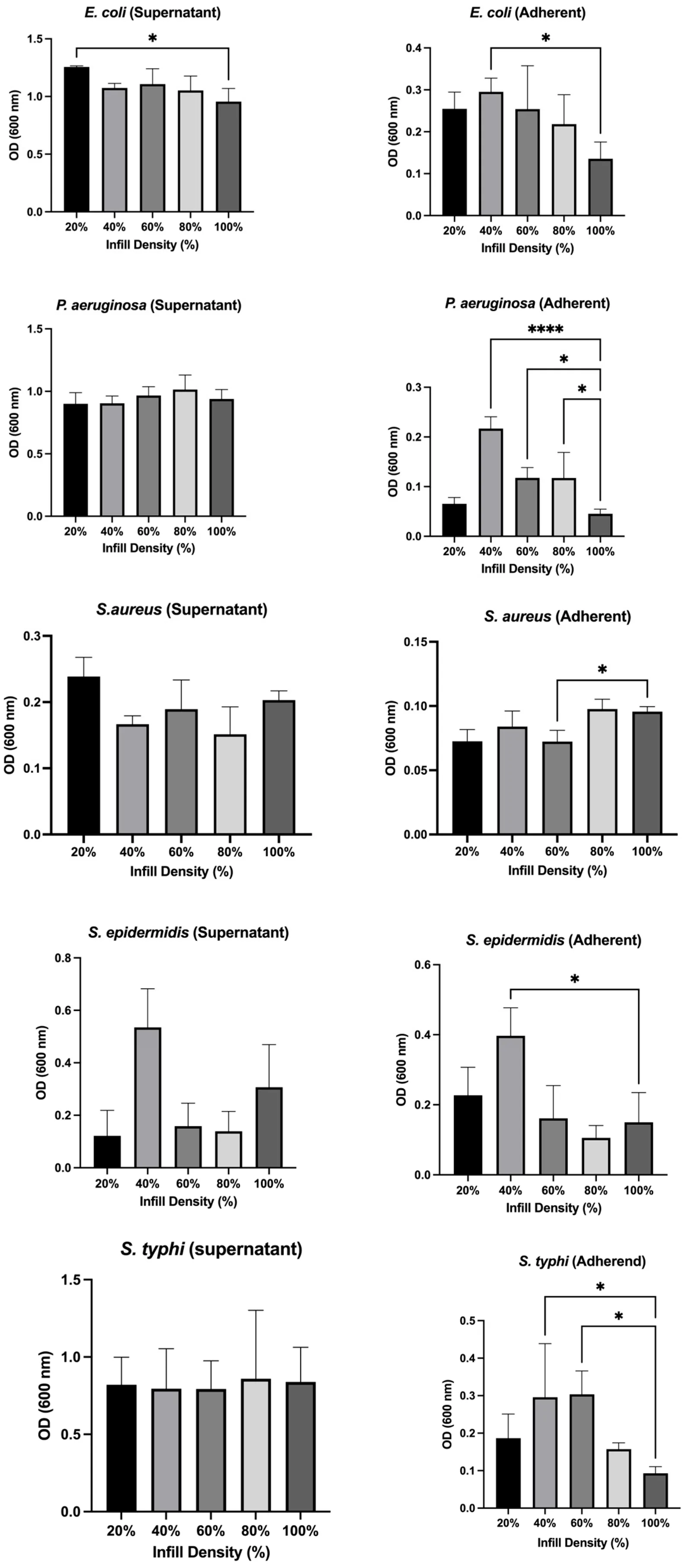
Effect of nominal infill density on planktonic and scaffold-associated bacterial biomass in 3D-printed PETG scaffolds. Five bacterial species (*E. coli*, *S. aureus*, *S. epidermidis*, *P. aeruginosa*, and *S. typhimurium*) were cultured in the presence of PETG scaffolds fabricated at nominal infill densities of 20%, 40%, 60%, 80%, and 100%. OD measurements were obtained for planktonic bacteria in the surrounding medium and adherent bacteria. The observed differences reflect architecture-associated variations in bacterial biomass and attachment and should not be interpreted as intrinsic antibacterial activity of PETG. Data are presented as mean ± SD (*n* = 3). Statistical analysis was performed using one-way ANOVA (* *p* < 0.05, **** *p* < 0.0001).

Figure 5 presents the optical density (OD) measurements of five bacterial strains cultured on 3D-printed PETG scaffolds at nominal infill densities of 20%, 40%, 60%, 80%, and 100%. Bacterial growth was quantified by measuring the OD of floating (planktonic) cells in the culture medium and adherent cells attached to the scaffold surface. The results demonstrated that the influence of infill density was species dependent and did not follow a uniform monotonic pattern across all microorganisms. *E. coli* exhibited relatively stable OD values in both the planktonic and scaffold-associated fractions across most infill-density conditions. However, significantly higher OD values were observed at 20% and 40% infill compared with 100% infill. Overall, these findings indicate that *E. coli* biomass and scaffold-associated attachment were only moderately affected by changes in PETG architecture, with the response being less pronounced than that observed for some of the other bacterial species. A more pronounced porosity-dependent response was observed for *P. aeruginosa*; scaffold-associated fraction decreased with increasing infill density, with the lowest value observed at 100% infill (*p* < 0.05). In contrast, no significant differences were detected in the planktonic fraction across the different infill-density groups. This suggests that changes in scaffold architecture had a greater influence on surface-associated *P. aeruginosa* than on the floating bacteria. For *S. aureus*, no significant differences were observed in planktonic OD across the tested infill densities. However, the scaffold-associated fraction showed significantly lower OD values at 60% and 100% infill, indicating that attachment was influenced by scaffold architecture in a non-monotonic manner. *S. epidermidis* showed architecture-dependent changes in both planktonic and scaffold-associated OD values. The lowest values were observed at the higher infill-density conditions, particularly 80% and 100% infill. However, the response was not strictly linear across all groups, indicating that the effect of scaffold architecture on S. epidermidis cannot be described simply as increasing or decreasing with infill density.

A similar distinction between planktonic and scaffold-associated responses was observed for *S. typhimurium*. No significant differences were detected in the planktonic fraction across the tested infill densities, whereas the scaffold-associated OD decreased at higher infill densities, with the lowest value observed at 100% infill. These findings indicate that infill density had a greater effect on scaffold-associated *S. typhimurium* than on planktonic biomass. Overall, the OD results demonstrate that PETG scaffold architecture influenced bacterial biomass and surface-associated attachment in a species-dependent manner. The responses were not uniformly monotonic across all organisms or infill-density conditions. In several species, the scaffold-associated fraction showed greater variation than the planktonic fraction, suggesting that printing-defined architecture primarily influenced bacteria–surface interactions rather than uniformly suppressing bacterial growth. Importantly, these changes should not be interpreted as evidence of intrinsic antibacterial activity of PETG, but rather as architecture-associated modulation of bacterial colonization.

**Figure 6:**
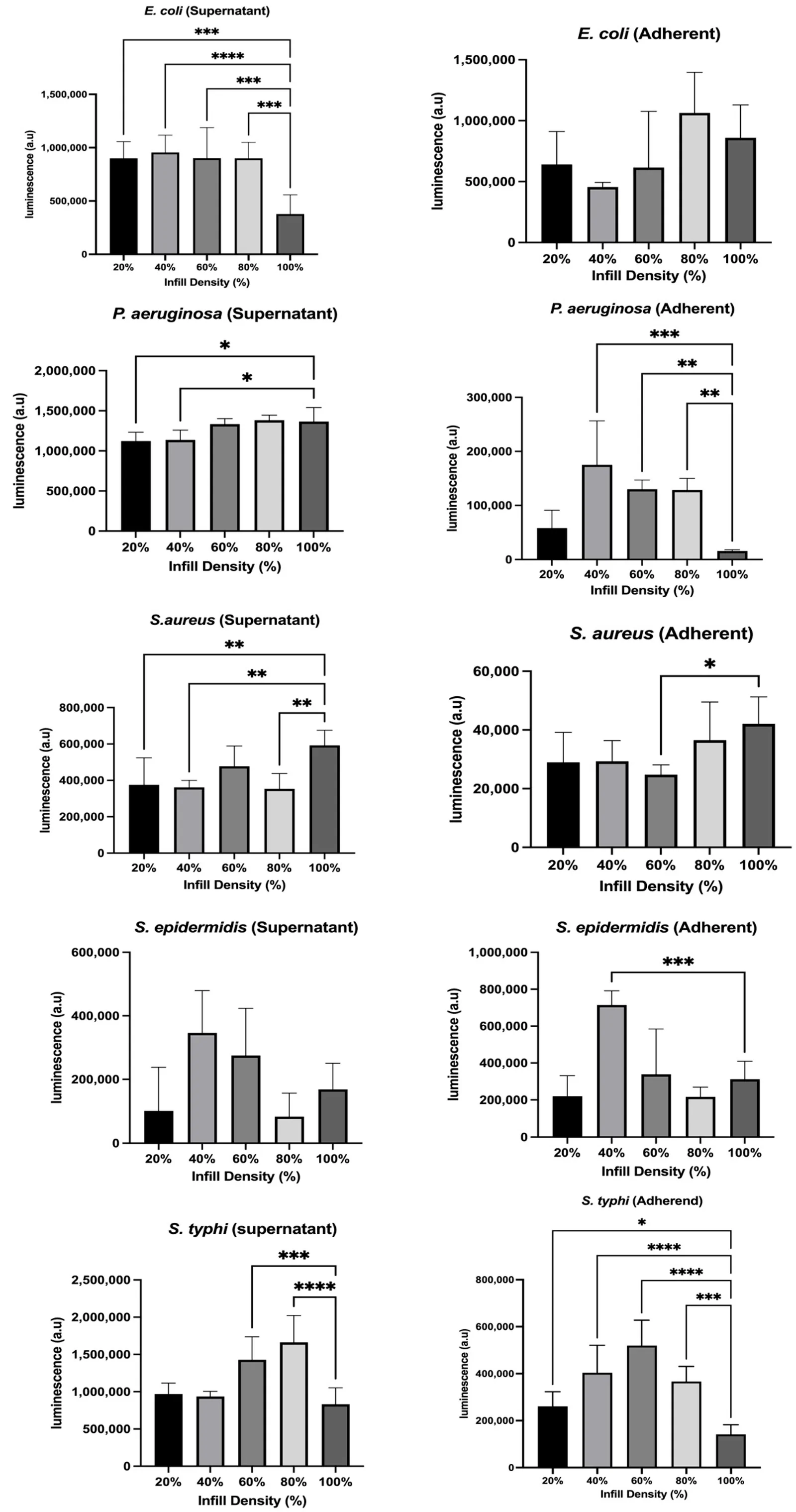
Effect of nominal infill density on ATP-based bacterial metabolic activity in planktonic and scaffold-associated fractions. ATP-associated luminescence was measured following incubation with PETG scaffolds fabricated at nominal infill densities of 20%, 40%, 60%, 80%, and 100%. Differences in ATP signal represent variations in metabolically active bacterial biomass under the tested conditions and should not be interpreted as evidence of intrinsic antibacterial activity of PETG. Data are presented as mean ± SD (*n* = 3). Statistical analysis was performed using one-way ANOVA (* *p* < 0.05, ** *p* < 0.01, *** *p* < 0.001, **** *p* < 0.0001).

Figure 6 presents ATP-based measurements of bacterial metabolic activity in the planktonic and scaffold-associated fractions following incubation with PETG scaffolds fabricated at different nominal infill densities. Overall, the results demonstrate that the effect of scaffold architecture on bacterial metabolic activity was species dependent and, in several cases, non-monotonic. Therefore, the ATP results are interpreted in terms of relative metabolic activity rather than as direct evidence of antibacterial activity or bacterial killing.

*E. coli* exhibited relatively stable ATP levels in both the planktonic and scaffold-associated fractions across the tested infill densities. Although minor variations were observed, no significant differences were detected in ATP activity for the scaffold-associated fraction. These results indicate that E. coli metabolic activity was comparatively less sensitive to changes in PETG infill architecture than that of some of the other bacterial species examined.

*P. aeruginosa* showed a more pronounced architecture-dependent response. ATP levels in the scaffold-associated fraction decreased as infill density increased, indicating lower metabolic activity of surface-associated bacteria at the higher infill-density conditions. In the planktonic fraction, ATP levels varied across the different groups without following a simple monotonic relationship. These findings demonstrate that the effect of PETG architecture on *P. aeruginosa* metabolic activity differed between planktonic and surface-associated populations. For *S. aureus*, scaffold-associated ATP levels were relatively similar at 20% and 40% infill but decreased at some of the intermediate and higher infill-density conditions, with the lowest value observed at 60% infill. ATP activity in the planktonic fraction also varied among the tested conditions. The absence of a consistent monotonic trend indicates that the metabolic response of *S. aureus* to scaffold architecture was dependent on the specific infill configuration rather than simply on increasing or decreasing infill density. For *S. epidermidis*, no significant differences were observed in ATP activity in the planktonic fraction, although a slight reduction was noted at some of the higher infill-density conditions. In contrast, the scaffold-associated fraction showed greater architecture-dependent variation, with lower ATP activity observed at higher infill densities, including a significant reduction at 100% infill compared with 40% infill. This indicates that scaffold architecture had a greater influence on surface-associated S. epidermidis metabolic activity than on the planktonic population. *S. typhimurium*, showed no significant differences in ATP activity were observed among the planktonic populations across the tested infill-density conditions. In contrast, the scaffold-associated fraction showed significant architecture-dependent differences, with higher ATP activity at some intermediate infill densities and lower activity at 100% infill. These findings further support the observation that PETG scaffold architecture exerted a greater influence on surface-associated bacterial activity than on planktonic populations.

**Figure 7:**
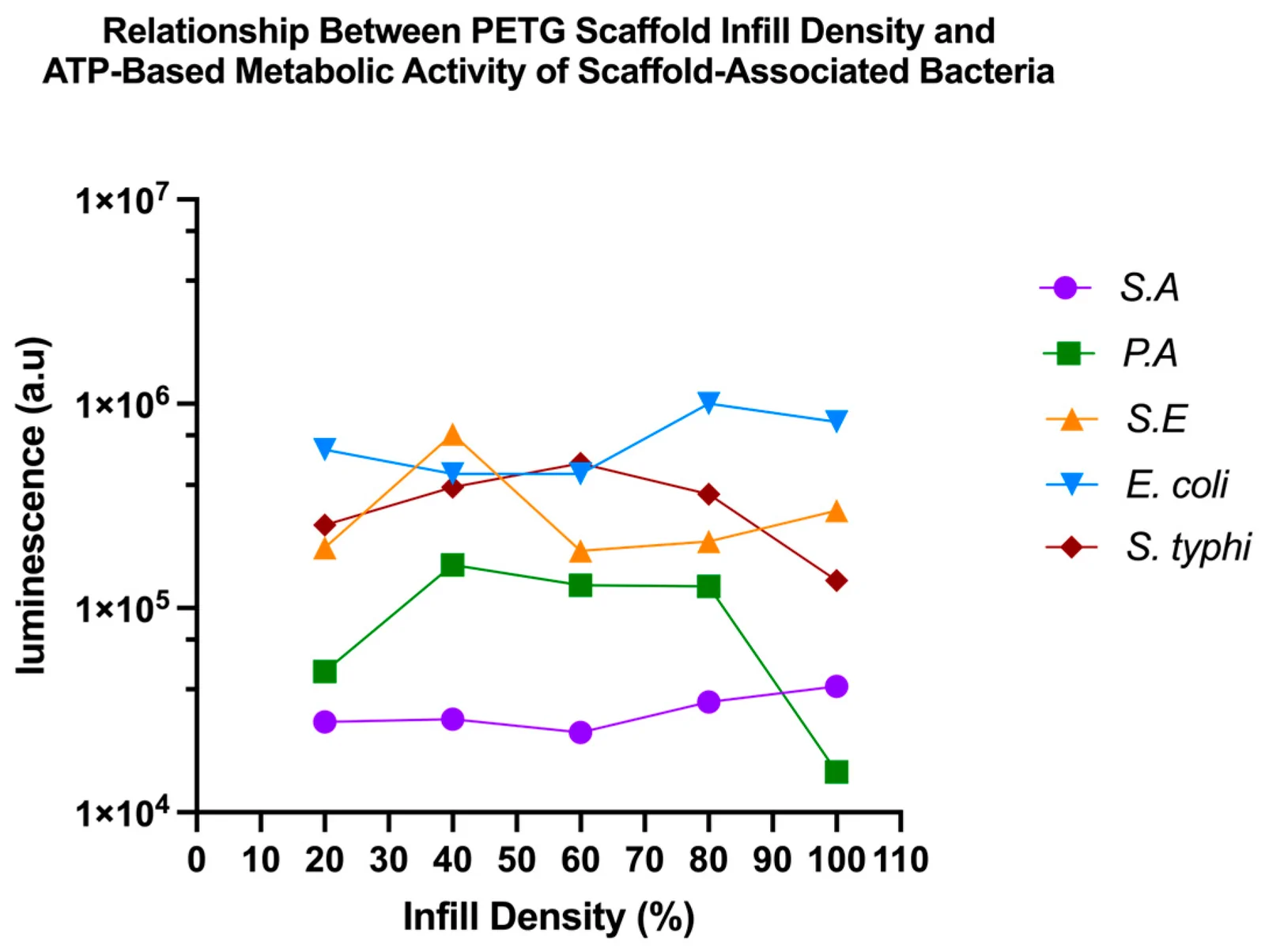
Relationship between nominal PETG infill density and ATP-based metabolic activity of scaffold-associated bacteria. Comparison of regression slopes showed no significant differences among bacterial species (F = 1.300, DFn = 4, DFd = 15, *p* = 0.3144), whereas regression elevations/intercepts differed significantly among species (F = 14.45, DFn = 4, DFd = 19, *p* < 0.0001), indicating species-dependent differences in overall ATP activity.

Linear regression analysis was performed to examine the relationship between nominal infill densityand ATP-based metabolic activity of scaffold-associated bacteria. Comparison of the regression slopes showed no significant differences among the five bacterial species (F = 1.300, DFn = 4, DFd = 15, *p* = 0.3144), indicating that the data did not provide evidence that the relationship between infill density and ATP activity differed significantly among species. In contrast, the regression elevations/intercepts differed significantly among bacterial species (F = 14.45, DFn = 4, DFd = 19, *p* < 0.0001), demonstrating significant species-dependent differences in overall ATP activity (Figure 7).

Taken together, the OD and ATP results demonstrate that PETG scaffold architecture modulated bacterial colonization and metabolic activity in a species-dependent manner. The direction and magnitude of these responses varied among bacterial species and were not consistently monotonic across the tested infill densities. In several cases, greater differences were observed in the scaffold-associated fraction than in the planktonic fraction, suggesting that infill architecture had a stronger influence on bacteria–surface interactions than on bacterial biomass in the surrounding medium. The findings therefore do not indicate intrinsic antibacterial activity of unmodified PETG. Rather, they demonstrate that changes in printing-defined architecture can alter bacterial attachment and metabolic behavior in a manner that depends on both bacterial species and infill configuration. These observations highlight infill density as an important design variable when considering bacterial colonization of 3D-printed PETG structures for biomedical and food-packaging applications.

Regression analysis was performed to further examine the relationship between nominal infill density and ATP activity of scaffold-associated bacteria. Comparison of the regression slopes showed no significant differences among the bacterial. This result indicates that the available data did not provide statistical evidence that the rate of change in ATP activity with infill density differed among species. However, this finding should not be interpreted as evidence that all bacterial species respond through an identical biological mechanism. In contrast, the regression elevations/intercepts differed significantly among species, demonstrating clear species-dependent differences in overall ATP activity. These differences are consistent with the distinct metabolic characteristics and surface-interaction behaviors of the bacterial species investigated. Accordingly, the regression analysis indicates that the bacterial species differed primarily in their overall levels of scaffold-associated metabolic activity, whereas no statistically significant differences were detected among the slopes describing their relationships with infill density. Overall, the regression findings support the species-dependent interpretation of the ATP data while avoiding the assumption of a universal antibacterial or mechanistic effect of PETG architecture. Infill density should therefore be considered an architectural factor capable of modulating bacterial metabolic activity rather than an intrinsic antibacterial property of the material.

The present findings are consistent with our previous study on 3D-printed PLA scaffolds, in which scaffold porosity significantly influenced bacterial attachment and microbial responses, demonstrating that pore architecture is a major determinant of microorganism–material interactions [21]. Likewise, Widjaya et al. reported that modifying the pore geometry (net diameter) of PETG carriers significantly altered biofilm attachment and microbial activity, confirming that scaffold geometry is a critical regulator of biofilm development [23]. This observation is supported by Al-Amshawee and Mohd Yunus, who identified carrier shape and pore size as major determinants of microbial immobilization, nutrient accessibility, biomass retention, and biofilm development, emphasizing that carrier geometry may be as important as the available surface area in regulating microbial colonization [24,25,26,27].

Porous structures have demonstrated that changes in scaffold porosity and thickness can alter bacterial proliferation, potentially through modifications in oxygen and nutrient transport within the scaffold architecture. Importantly, these effects may be species dependent, indicating that scaffold architecture does not simply increase or decrease bacterial colonization uniformly but instead creates microenvironments that differentially influence microbial attachment and physiological activity [26,28,29].

Together, these findings support our observation that PETG scaffold architecture can regulate both bacterial attachment and metabolic activity by modifying the available attachment area, nutrient transport, and bacteria–surface interactions [10,11,30].

The influence of scaffold architecture on bacterial behavior is further supported by previous investigations of PETG-based biomaterials. Structural characteristics such as porosity, surface roughness, and printing resolution have been shown to regulate biological interactions on polymeric surfaces [15]. For example, Shilov et al. demonstrated that the surface properties of 3D-printed PETG structures significantly influence cellular adhesion and material–cell interactions, highlighting the importance of surface architecture in governing biological responses [14]. Similarly, Daskalakis et al. reported that porous PETG scaffolds fabricated by additive manufacturing exhibit increased surface roughness and interconnected porosity, features that can substantially alter biological interactions, including microbial colonization [17]. Together with the present findings, these studies provide compelling evidence that scaffold architecture is a primary determinant of bacterial attachment, biofilm formation, and metabolic activity on PETG materials. Optimizing pore architecture therefore represents a promising strategy for developing PETG scaffolds with improved resistance to bacterial colonization while maintaining the structural and functional properties required for biomedical and food-packaging applications [16,31,32].

## 4. Conclusions

This study demonstrates that the nominal infill density of 3D-printed PETG scaffolds is a key design parameter influencing both their mechanical performance and bacterial interactions. Tensile testing revealed that increasing infill density resulted in a progressively higher ultimate tensile strength and Young’s modulus, whereas lower infill densities were associated with reduced mechanical strength and greater deformation at failure. These findings confirm the expected trade-off between material content, internal architecture, and mechanical performance.

The microbiological results further demonstrated that the effect of scaffold architecture influenced bacterial growth, attachment, and metabolic activity was in species dependent manner. Optical density and ATP-based assays indicated that changes in infill density generally had a greater influence on scaffold-associated bacteria than on planktonic populations. *E. coli* showed relatively limited variation across the tested infill conditions, whereas *P. aeruginosa*, *S. epidermidis*, and *S. typhimurium* exhibited more pronounced architecture-dependent changes in scaffold-associated biomass and/or metabolic activity. *S. aureus* also showed variable responses across the different infill-density conditions without a consistent monotonic trend. Importantly, these findings should not be interpreted as evidence of intrinsic antibacterial activity of unmodified PETG; rather, they indicate that printing-defined architecture can modulate bacterial attachment and metabolic behaviour.

Overall, the findings highlight the importance of optimizing PETG infill architecture to achieve an appropriate balance between mechanical performance and susceptibility to bacterial colonization. Because actual scaffold porosity was not independently measured in the present study, the results are interpreted in relation to nominal infill density rather than absolute porosity. These findings may support the rational design of PETG structures for biomedical and food-packaging applications, where both structural reliability and control of surface-associated bacterial colonization are important. Future studies should directly quantify actual porosity and surface morphology and incorporate complementary imaging techniques to further clarify the relationship between scaffold architecture and bacterial colonization. The incorporation of antimicrobial agents may also be explored as a separate strategy to provide true antibacterial functionality while maintaining the required mechanical properties.

## Figures and Tables

**Table 1:** Tensile properties of 3D-printed PETG samples fabricated at different nominal infill densities.

Nominal Infill Density (%)	UTS (MPa)	Young’s Modulus (MPa)	Strain at Fracture (%)
**20**	1.27 ± 0.15	18.5 ± 3.7	74.0 ± 11.1
**40**	2.79 ± 0.28	47.9 ± 8.1	53.7 ± 6.4
**60**	5.72 ± 0.46	133.6 ± 18.7	51.8 ± 6.2
**80**	8.54 ± 0.60	224.7 ± 27.0	56.5 ± 6.8
**100**	12.50 ± 0.75	418.0 ± 41.8	31.9 ± 3.2

## Data Availability

The original contributions presented in the study are included in the article, further inquiries can be directed to the corresponding author.

## Abbreviations

The following abbreviations are used in this manuscript:

3D	Three dimensional
ASTM	American Society for Testing and Materials
ATP	Adenosine triphosphate
FDM	Fused deposition modelling
FFF	Fused filament fabrication
OD	Optical density
PETG	Polyethylene terephthalate glycol
UTS	Ultimate tensile strength
